# The Effects of Early Physiotherapy Treatment on Musculoskeletal Injury Outcomes in Military Personnel: A Narrative Review

**DOI:** 10.3390/ijerph192013416

**Published:** 2022-10-17

**Authors:** Patrick Campbell, Rodney Pope, Vinicius Simas, Elisa Canetti, Benjamin Schram, Robin Orr

**Affiliations:** 1 Tactical Research Unit, Bond University, Robina, QLD 4229, Australia; 2 School of Allied Health, Exercise and Sports Sciences, Charles Sturt University, Albury, NSW 2640, Australia; 3 Faculty of Health Science and Medicine, Bond University, Robina, QLD 4229, Australia

**Keywords:** early treatment, delayed treatment, musculoskeletal injury, military, occupational, physical therapy, physiotherapy

## Abstract

The risks and incidence rates of musculoskeletal injuries among military personnel are high, and the importance of physiotherapy in treating these injuries is well established. However, what is less clear is whether the timing of commencement of physiotherapy treatment affects musculoskeletal injury outcomes in military personnel. This lack of clarity is exacerbated by the known underreporting of injuries among military personnel, and the resulting self-management of musculoskeletal injuries using analgesics, non-steroidal anti-inflammatories and other means. This narrative review was designed to identify and synthesize current evidence regarding the effects of timing of physiotherapy treatment on musculoskeletal injury outcomes, focusing on potential benefits of early versus typical or delayed commencement of physiotherapy treatment. Overall, current evidence suggests early physiotherapy treatment of musculoskeletal injuries offers distinct advantages over typical or delayed commencement of physiotherapy treatment in military settings. Specifically, it appears early treatment expedites recovery in early phases following injury onset and benefits longer term mental health and well-being. It may also reduce the need for more invasive and costly health care interventions and enable earlier return to training and operational service. Importantly, a cultural shift within military contexts to ensure early reporting of musculoskeletal injuries is required if the benefits of early commencement of physiotherapy treatment are to be achieved.

## 1. Introduction

Military personnel are exposed to a range of occupational risk factors which may result in, or contribute to, an array of musculoskeletal injuries (MSI), illnesses, wounds, disabilities, and other conditions. Many of these conditions will require initial and ongoing medical care, once they have occurred, and controlling the factors that lead to them can be challenging in military contexts. For example, military training involves cumulative bouts of exposures to high-volume physical training, acutely intense high-impact physical demands, and combat and noncombat training drills [1,2,3]. Additionally, military equipment demands, such as the use of body armor and combat ensembles which can weigh over 45 kg [1,4], contribute to mobility deficits [5] and limitations in task performance and mission capabilities [6], and increase the likelihood of occurrence of MSI [7]. As a result of the exposure of personnel to intense physical demands and military-specific occupational requirements, the risks and incidence rates of MSI in military environments are high [8]. While MSI rates vary substantially by injury type, previous data have shown an overall incidence rate for reported injuries of 628 injuries per 1000 person-years in the U.S. Army [9], with over half of U.S. Army soldiers seeking treatment for MSI within a 12-month period [10]. Sprains and strains and overuse injuries, like stress fractures, are the most commonly experienced categories of MSI in military environments [11,12]. The high burden of MSI translates to MSI being the primary factor contributing to disability, medical care, and losses of operational effectiveness in combat zones, and MSI comprising the leading cause of limited duty days [8,10,12,13,14,15]. Pope et al. [16] found that Australian Army recruits who experienced a lower limb MSI were 10 times as likely to fail to complete training and be discharged as peers who did not sustain a lower limb MSI. In the U.S. military, Holsteen et al. [17] found that lower limb MSI resulted in 13% of all military service days being restricted to limited duties, with 67% of those limited duty days due to permanent limitations of duties in individual personnel resulting from lower limb MSI. These figures indicate that military capability is substantially degraded on any given day due to MSI and that risk of discharge is heightened following MSI. Despite large volumes of evidence evaluating the extent of the problem of MSI within the military and the identification of various risk factors for MSI, there is little evidence that interventions have led to sustained reductions in the incidence of MSI and the burden MSI place on military health systems [10].

Similarly, while enhancements to technology, military equipment, and physical training have been, and continue to be, made [18,19], military-specific occupational and training related factors contributing to MSI (e.g., specific military training, load carriage, combat activities) are often unavoidable requirements of military service, and, as such, comprise a large and difficult-to-address contributor to occurrence of occupational MSI. For these reasons, it is worth considering different avenues for improving health outcomes; not just limiting or modifying exposures to risk factors of MSI, but also the management and treatment of resulting injuries. 

Physiotherapy (also known as physical therapy) is a well-established avenue for providing early treatment to diagnosed or suspected MSI in military forces [20]. Physiotherapy is an effective, evidence-based branch of allied health services with demonstrated efficacy in treating and managing MSI [20,21,22,23]. The implementation of clinical referrals to physiotherapy has been reported to be a cost-effective approach resulting in accelerated times to recovery, earlier return to work, lessened pain symptoms, and reduced incidence of invasive and costly medical procedures and diagnostics [20,21,23,24,25]. Available physiotherapy treatment strategies and guidelines often involve the use of loaded and unloaded muscular strength and endurance exercises, joint stabilization exercises, injury and rehabilitation education, and manual therapy techniques [23,26]. Overall, there is a considerable breadth of evidence to support referral to, and involvement of, physiotherapy in the management of MSI. Within military health systems, specifically, physiotherapists have been reported to have very high levels of knowledge regarding how to manage MSI in military personnel [20]. As a result, there is strong evidence to support the role of physiotherapy in military health systems, to: reduce the injury burden; improve service member patient outcomes; achieve reductions in down-stream, more expensive and invasive health care utilization (e.g., further medical diagnostics, surgeries); reduce the development of chronic pain and disability; and, ultimately, enable greater retention of personnel and increased and sustained operational effectiveness [8,10,13,14,15,27]. 

Noting the likely importance of early physiotherapy treatment of MSI, it must also be recognized that there may be barriers to early intervention approaches, given prevailing attitudes within military populations and health systems. Firstly, there is a propensity for military personnel to delay or avoid reporting MSI [28,29,30]. Several reasons for this reticence of serving members to reporting MSI in the Australian Defence Force (ADF) have been reported including, but not limited to, a pervading culture in the military of perseverance and toughness, and concern that reporting an MSI could adversely impact on a member’s prospects of deployment or, in extreme cases, result in their discharge from the military [29,31]. The resulting under reporting is noted in a report on osteoarthritis in the lower limbs produced by Bond University and commissioned by the Australian Department of Veterans’ Affairs whereby up to 192 of 393 injuries per 100 full-time equivalent years of service were estimated to not be reported [30]. Examples drawn from the U.S. Army highlight research findings indicating 64% of Army trainees and 58% of an Infantry Brigade Team did not disclose they had sustained an injury when in fact they had [27,28]. Concomitant to this delayed or under reporting, research suggests that soldiers frequently try to self-manage their injuries [27,28,30]. For example, despite the high capacity for good quality care within military health systems, a study by Sauers et al. [27] found respondents from a U.S. Infantry Brigade indicated that they generally self-managed the many injuries they did not report, most often using over-the-counter pain medication (81%), ice packs (55%), heat packs (52%), hot tub immersion (40%), pain avoidance (37%), splints or braces (25%), or a range of less common approaches, including (in descending order of frequency) yoga, narcotics, meditation, topical/muscle rubs, illicit drugs, additional sleep, massage, and alcohol. 

A second barrier to early treatment of MSI is the currently very limited evidence-base for earlier health care interventions across the broad range of MSI conditions. While there is strong evidence for utilization of physiotherapy, for example, as a more conservative treatment option, and therefore avoiding over-medicalization of MSI [22,26], the optimal timing of commencement of physiotherapy interventions is less well researched. Much of the evidence on the effects of early physiotherapy interventions on injury outcomes has been derived from back (or spinal) pain research [22,26,32,33,34,35,36,37,38,39,40,41,42]. Consequently, there is less evidence evaluating early physiotherapy treatment in other musculoskeletal conditions. 

Therefore, while the available evidence clearly identifies the acute and chronic burdens of MSI impacting military health systems and serving members, the existing literature is less clear in relation to the optimal timing of physiotherapy treatment across a range of MSI conditions. Consideration is also needed of the potential impacts of military-specific cultural factors, when making any guideline recommendations regarding early physiotherapy treatment of MSI in military populations. On this basis, the aims of this narrative review were: to explore the existing evidence evaluating the effects of timing of delivery of physiotherapy treatment on patient outcomes; and to provide an exploratory narrative to consider potential links between timing of physiotherapy treatment delivery in military-specific environments and potential impacts of the treatment on personnel health, organizational costs, and operational effectiveness of military forces.

## 2. Impacts of Timing of Physiotherapy Care

As noted above, there is considerable evidence demonstrating the efficacy of physiotherapy as a key component of treating and managing MSI [24,35,43]. This is reflected in the inclusion of physiotherapy in treatment guidelines for common conditions such as ankle sprains [44] and lower back pain [21]. Interestingly, referral to, or inclusion of, physiotherapy in treatment protocols has been recommended to be delayed in both of these commonly encountered injuries [21,44]. This recommended delay in commencement of physiotherapy care for these conditions was largely due to the spontaneous recovery of low back pain [45], and interactions with inflammatory processes (e.g., pain or swelling) following an ankle sprain [44]. While there are recommendations for the inclusion of physiotherapy within guidelines for management of some injury conditions (e.g., knee osteoarthritis, ankle sprain, lower back pain) [44,45,46] and for specific physiotherapy treatment types (e.g., manual therapy, muscle strengthening, joint mobilizations) [23,26,44], there is a surprising scarcity of evidence-informed recommendations relating to the timing of that treatment in conditions other than ankle and spine-related injuries. There are several potential methodological challenges for studying the effects of treatment timing, and many of the studies which have evaluated physiotherapy treatment timing effects, to date, suffer from these challenges and associated limitations. For example, there are definitional issues and subsequent variations across studies in defining what constitutes ‘early’ and ‘delayed’ treatment. There are also substantial risks of bias due to potential confounding arising from methods of patient allocation to treatment types. This may explain the scarcity of relevant literature, and these methodological limitations will be discussed later in this review. 

### 2.1. Evidence from Systematic Reviews

Current evidence on the timing of physiotherapy treatment and its impacts on outcomes for patients with back pain largely suggests equivalent longer-term outcomes, regardless of when treatment commenced. However, much of the evidence demonstrates earlier physiotherapy accelerates initial recovery from pain symptoms or facilitates faster return to a state of physical recovery [21,23,26,47]. For example, four recent systematic reviews have examined the influence of timing of commencement of physiotherapy treatment on patient outcomes [21,23,26,47]. Of these systematic reviews, two included only neck or back pain studies [21,26], and one focused on chronic pain [47], with 82% of the studies included in the fourth review also focusing on neck or back injuries [23]. Overall, the findings of these systematic reviews have indicated there is low quality evidence supporting the use of early physiotherapy treatment to reduce total health services utilization costs [21,23,26]. The evidence concerning the impacts of early physiotherapy treatment versus delayed or usual care on clinical patient outcomes was less clear; together, results indicated that, while there were no negative effects of early physiotherapy care, there was, overall, mixed evidence regarding patient clinical outcomes from such care [21,23,26]. Specifically, Ojha et al. [21] identified evidence that supported a dose–response relationship between early physiotherapy treatment and reductions in health services utilization costs relating to services such as medical imaging, medication supply (including opioids), and surgeries for patients with spinal-related pain conditions. While the other systematic reviews did not specifically state a dose–response relationship was present, based on synthesis of findings from included studies, their conclusions indicated consistent evidence for reductions in health services utilization when early, rather than delayed, physiotherapy management of injuries occurred [23,26]. However, both Arnold et al. [26] and Deslauriers et al. [23] found the differences in health services utilization less clear when examining early versus typical timing of physiotherapy injury management. 

Findings relating to clinical outcomes such as quality of life, pain and disability for patients receiving early physiotherapy treatment were again inconclusive when they were compared to the same types of outcomes in groups that received usual or typical timing of physiotherapy care [21,23,26]. However, early commencement of physiotherapy treatment yielded beneficial improvements when compared to delayed treatment [21,23,26]. 

Importantly, there were very few randomized controlled trials or prospective cohort studies included in any of these cited systematic reviews, with the evidence mostly derived from retrospective cohort studies [21,23,26]. This limits the conclusions that can be drawn and any potential recommendations based upon those reviews. The evidence was also mostly drawn from studies conducted in the general population, with participants drawn from community-based samples, workers compensation databases, or general practices [33,34,35,36,48]. There were very few studies which included military cohorts [22,49,50], or populations that may be considered similar to military populations; thus adding additional limitations to the evidence base. 

### 2.2. Evidence from Primary Studies

Table 1 primarily lists the characteristics and key findings of studies included in the four systematic reviews detailed above that were relevant to the aims of the narrative synthesis presented here; and also includes details of some additional primary studies. Within the studies listed in Table 1, there were several evaluating the use of early compared to usual or delayed commencement of physiotherapy treatment for MSI. These highlighted several pertinent beneficial effects of early access to physiotherapy treatment, as well as limitations within the literature. For example, multiple RCTs have shown greater improvements at certain follow-up time-points in various outcomes (e.g., pain, disability, general health, etc.), occur with early physiotherapy treatment when compared to either usual care or delayed care for tennis elbow, and neck or lower back pain [33,37,38,39,51]. However, another RCT examining the effect of early physiotherapy intervention when compared to delayed intervention on whiplash disorders showed no differences in outcomes between the two groups [40]. There are several interesting methodological considerations related to the interactions between outcome variables and follow-up time-points which indicate the evidence is less certain and more complex than it might appear from some studies, in regard to reported benefits of early access to physiotherapy treatment. Four studies [33,37,38,51] showed initial greater improvements with early physiotherapy treatment at earlier time-points (varying between 1 and 3 months), but at longer term follow-up (varying between 6 and 12 months) the differences between the early care groups and the usual or standard or delayed care groups was minimal, clinically not meaningful, or non-significant. Therefore, overall while there were initial greater improvements in measured outcome variables associated with early physiotherapy care, findings from these RCTs seem to indicate that the improvements in patient outcomes tend to reduce, with outcomes becoming more similar between groups, as time from injury onset progresses. 

In a study of physiotherapy care which may have important implications for military personnel, Wand et al. [38] showed that while there were no meaningful differences in physical clinical outcomes between early and delayed care groups at 3 and 6 month time-points post-injury, patients in the early care group had reduced anxiety, depressive symptoms, and distress at longer term follow-up. Additionally, Childs and colleagues [22] found early physiotherapy treatment was associated with less use of opioid medications. Given the documented challenges military veterans experience with pain, opioid use, mental health [31], and general health and wellbeing [52,53,54] following their service periods, and the associations between injury and discharge or attrition [16,55], these findings may have important implications for military organizations to consider when decisions are made regarding timing of physiotherapy treatment. They may also have important implications when military organizations are considering encouraging and cultivating cultures that support military personnel to seek early treatment and report earlier any symptoms of MSI. 

There is also additional evidence which may further support the use of early physiotherapy treatment interventions in military settings, specifically, despite this evidence being derived from lower-quality research designs than the RCTs mentioned above. For example, Childs et al. [22] found early physiotherapy for lower back pain in a military population was associated with reduced use of medical imaging, spinal injections, spinal surgeries, and opioids prescribed for pain treatment. Young et al. [49] also examined the effect of timing of physiotherapy treatment for patellofemoral pain within the military health system, and found individuals who received early treatment (<30 days after diagnosis) had lower recurrence rates of injury and reduced on-going medical costs and healthcare utilization (e.g., medical imaging, medication use, or injections) than those whose treatment was delayed. Similar findings were recently published by Rhon et al. [50], who found the odds of ankle sprain recurrence were greater with delayed access to rehabilitation (i.e., care at 8–12 weeks) within the U.S. Military Health System, when compared to earlier access to treatment (i.e., within 4 weeks). Additionally, individuals who had delayed care required a greater number of total rehabilitation visits, and a linear increase in cost of care was observed with each day rehabilitation was delayed [50]. 

Together, the findings from these studies clearly demonstrate positive outcomes to be associated with early access to physiotherapy care within a military context, specifically, and comprise the very limited evidence from military contexts available to draw from. In other contexts, three studies also demonstrated that early access to physiotherapy treatment in civilian workers was associated with faster return to work and fewer numbers of restricted workdays, when compared to delayed access [41,42,56]. Workers’ compensation and insurance data considered in other studies also indicate that delays in reporting injuries, receiving medical care and/or initiating work-related disability care are associated with increased durations of temporary disability and higher medical costs [57,58]. Translated into a military context, early intervention could then be considered to lead to increases in operational effectiveness and reduced costs due to less medical interventions, reduced medical costs, lower numbers of restricted training or workdays, and reductions in use of potentially harmful pain medications. 

## 3. Discussion

Overall, evidence regarding the benefits or risks associated with timing of the commencement of physiotherapy treatment for MSI is complex and somewhat unclear due to the varying contextual factors contributing to each study design. However, there seems to be substantial evidence suggesting initial accelerated improvements in patient outcomes in early treatment groups when compared to usual or delayed care groups. Whether these positive effects of early treatment remain evident at time points of follow up that are months and years subsequent to injury onset is unclear and may vary with injury type, with many studies demonstrating a convergence of measured outcomes across early and delayed physiotherapy treatment groups from 6–12 months. In summary, while the evidence from RCTs may be indicating that earlier physiotherapy treatment reduces pain, medical imaging use and disability, and improves perceived quality of life, these patient outcomes all likely converge across groups, with differences between early and delayed treatment groups becoming minimal at later time-points following initial onset of injury. However, in military settings the observed early positive effects of early physiotherapy treatment may be magnified due to the demanding natures and contexts of military occupations. As a consequence, what may be seemingly non-meaningful early benefits for civilians, when longer term convergence in outcomes across groups and wait-times in public health systems are considered [23], may actually be of critical importance in military contexts. If accelerated times to recovery can be achieved through early physiotherapy care, this may confer substantial benefits to the military organization in terms of its operational effectiveness and reduced costs. Losses in training or work time are likely to be particularly consequential in military settings due to the need for ongoing training to maintain operational readiness, and the need to have personnel fit and able to deploy to ensure operational effectiveness. On this basis, the observed early benefits of early access to physiotherapy may be more highly valued in these military settings than in the general population, even if individuals end up with similar long-term outcomes. As such, it is not surprising that physiotherapy intervention is considered a ‘force multiplier’ for military organizations [60].

These effects of early access to physiotherapy treatment may couple with opportunities that arise from those to achieve earlier restoration of physical and/or physiological qualities, to yield even greater benefits for military personnel recovering and returning from MSI. This is particularly so, given the occupational demands of military personnel compared to those commonly required of civilian populations. For example, evidence has indicated that even among civilians, only those patients who received early physiotherapy treatment after MSI had reattained similar range of motion at the site of injury, at a three-year follow-up, to that evident in uninjured individuals [39]. This may be even more important for personnel, like military personnel, who are in occupations that require higher levels of physical function, since their ability to work and deploy is often contingent on them demonstrating sufficient fitness and functional capacity. Failure to reattain normal joint range of motion and functional capacity for job roles may also have down-stream effects on costs to military health services and veteran agencies due to injuries sustained while in service. Taken together, these considerations mean there may be great importance in military personnel gaining access to early physiotherapy treatment in order to restore previous levels of physical function. However, we acknowledge these likely impacts cannot be verified at this current time due to the lack of military-specific studies on this topic, with most of the currently available evidence drawn from civilian populations [21,23,26,33,34,35,36,48].

The influence of timing of physiotherapy treatment on development of secondary adverse outcomes, such as potential for weight gain and reductions in physical fitness which arise from MSI, are not well-described within the current literature. Indeed, there appears to be a dearth of evidence describing such second-order effects within studies which examine differences in patient outcomes between early treatment and delayed or usual care groups. However, there is evidence from separate studies that experiencing MSI is associated with weight gain and decreases in fitness [61,62,63]. These factors may then further exacerbate injury risk beyond the risk arising from prior injury alone [64], as increased weight or overweight and obesity, as well as reduced fitness levels and reductions in physical training, have also all been associated with increased risk of MSI in military populations [16,65,66]. As a consequence, it is reasonable to hypothesize that participation in early physiotherapy treatment, which may accelerate recovery and bring forward interventions that address these additional factors, may lead to reduced losses in fitness and associated weight gain. The observed lower rates of recurrence of patellofemoral pain within military personnel who undertook early treatment (compared to delayed), in the study conducted by Young et al. [49], may constitute preliminary evidence of this phenomenon occurring. However, future research should examine these additional outcomes, given their importance to military organizations in terms of operational effectiveness and health of personnel. 

### Limitations

Substantial conceptual problems within the literature, relating to the definitions of early treatment and what constitutes ‘early’ versus ‘delayed’, were also noted in this review. There is significant variation in the timeframes termed ‘early’ within the studies outlined in this review, with ‘early’ encompassing periods ranging from immediate up to 28 days following an initial visit to physician or physiotherapist [22,32,33,34,35,36,37,38,39,40,41,42,48,51,56,59]. Similar variation was observed in what constituted ‘delayed’ within the literature. It is clearly difficult to develop clear understandings and synthesize findings from primary studies when there is such considerable variation around key concepts relating to the research question. As such, we strongly recommend common definitions are developed regarding the concepts of early and delayed treatment, with the lack of standardization representing a clear limitation within the current body of evidence. Additionally, this review included studies both with a clear and definitive musculoskeletal injury diagnosis (i.e., positive imaging scans, presence of pain and positive signs on physical examination) and studies of musculoskeletal pain symptoms in individuals receiving treatment for that pain. Future studies and reviews may want to consider the possibility of that outcomes of early or delayed physiotherapy treatment may differ between diagnosed MSI and pain symptoms without a definitive diagnosis. This review and the evidence it considered were also limited by a lack of studies that have directly investigated military populations. For example, the majority of studies examined throughout the review in relation to timing of treatment drew on populations within a civilian context and comprised lower back and neck pain injuries. 

However, we have provided a narrative that sought to draw conclusions based on extrapolation of findings of research conducted in the general population to military personnel. We hypothesize that future studies are likely to show greater benefits to short- and long-term outcomes of early versus delayed or standard treatment, in both military personnel and military organizations-when compared to the general population-due to the occupational demands and contexts of military roles. Such future studies, when conducted, should preferably be RCTs, as there are considerable risks of bias and confounding associated with patient selection in studies comparing outcomes between cohorts formed in non-random ways (e.g., risks of bias and confounding arising from variability in levels of self-efficacy, psychosocial differences, and psychological differences). 

## 4. Conclusions

This narrative review has shown that, while there is not clear evidence for longer-term benefits of early physiotherapy treatment for MSI, substantial evidence supports earlier benefits of early treatment. These earlier benefits of early injury management may be particularly important for military personnel. The synthesis of findings provided above suggests the initial beneficial effects of exposure to early physiotherapy following MSI may translate into an accelerated recovery and return to duty for military personnel, and may reduce the number of limited duty days personnel experience-a documented concern in military organizations. Ultimately, these early benefits of early access to physiotherapy may culminate in an increase in the operational effectiveness of military organizations if there are less limited duty days, and personnel experience more rapid recovery in the early phases following injury, which allow an earlier return to normal duties and readiness for deployment. This is so, even if the longer-term outcomes are similar between those receiving early and usual or delayed treatment. The positive effects of early physiotherapy treatment may be further enhanced by flow-on benefits for additional outcomes of importance, with some preliminary evidence indicating lower opioid use and mental health issues (anxiety, depressive symptoms and distress) at later time-points following MSI in those receiving early physiotherapy. These further, longer-term benefits may translate to reduced costs associated with managing veterans’ injuries or health conditions. However, such additional, long-term benefits were not demonstrated in all studies. 

Future research should determine if these sorts of effects can be confirmed through RCTs. Non-randomized study designs investigating early vs. usual or delayed treatment are likely to be prone to confounders that will inhibit the ability to draw strong conclusions regarding the efficacy of early physiotherapy management approaches. Overall, it is likely the utilization of early physiotherapy treatment of MSI offers distinct advantages over usual or typical treatment approaches in military settings, particularly in expediting recovery and return to physical conditioning activities in early phases following injury onset but possibly also for longer term mental health and well-being. It may also reduce the need for more invasive and costly health care interventions—for example, medical imaging and surgery. These findings are evidence of the importance of efforts to establish positive cultural beliefs in accessing early care for musculoskeletal injuries and complaints in military environments, and should be cited as a basis for facilitating cultural change. 

## Figures and Tables

**Table 1:** Characteristics and key findings of primary studies evaluating outcomes of early and delayed musculoskeletal injury treatment.

Study	Study Design	Participant Demographics	Definition of Early and Delayed Access to Treatment or Care	Summary of Key Findings
Childs et al. [22]	Retrospective cohort	Participants accessing the U.S. Military Health System (*N* = 753,450; age range = 18–60 years) for lower back pain (LBP)	**Early access**: Physical therapy visit occurring within 14 days of primary care index date visit **Delayed access**: Physical therapy visit occurring between 14–90 days after primary care index date visit	Early access participants who were adherent to physical therapy protocols had significantly lower healthcare use, including lower use of advanced imaging, lumbar spinal injections, lumbar spine surgery, and opioid medications, and lower total LBP-related costs *. * Compared to early (nonadherent), delayed (adherent), and delayed (nonadherent) participants
Ehrmann-Feldman et al. [42]	Prospective cohort	Workers’ compensation cohort from Quebec (Canada) experiencing back injuries (*N* = 2147)	**Early access:** Workers receiving physical therapy within 30 days of the injury **Delayed access:** Workers not receiving, or never referred to physical therapy, or referred more than 30 days following the injury	Receiving early access (i.e., within 30 days) to physical therapy reduced the odds of having an absence from work of more than 60 days following back injury when compared to not receiving early access to physical therapy (aOR = 0.13, 95% CI 0.06–0.3).
Fritz et al. [33]	Randomized Controlled Trial	Patients (general population) with lower back pain attending a primary care physician (*N* = 220; age range = 18–60 years).	**Early physical therapy**: Patients commenced treatment within 72 h of study enrolment and received four physical therapy sessions within the initial 4 weeks **Usual care**: Patients received education on back pain and a resource providing advice consistent with lower back pain guidelines. No further intervention received.	Those receiving early physical therapy had a statistically significant improvement in the Oswestry Disability Index (ODI) at four weeks and at 3 months, but not at 1 year, when compared to those receiving usual care. However, the level of improvement in the early physical therapy group did not reach minimum clinically important differences when compared to levels associated with usual care. Secondary outcomes of the study were mixed, with early care not showing benefits at follow-up time points for pain intensity, physical activity outcomes, and quality of life scales; while patient-reported success and overall health, and fear-avoidance beliefs scales showed significant improvements in the early care group when compared to usual care. There were no differences between groups at follow-up for health care utilisation.
Fritz et al. [36]	Retrospective cohort	Patient (general population) data retrieved from U.S. database of employer-sponsored healthcare plans (*N* = 32,070), inclusive of new primary care low back pain consultations. Note: patients enrolled in comparative groups share similar population demographic characteristics, index diagnoses, comorbidities, hospitalisations, and/or narcotic use.	**Early access**: Patients received physical therapy treatment ≤ 14 days following primary care index date. **Delayed access**: Patients received physical therapy treatment between 15 and 90 days following primary care index date.	Access to early physical therapy referral was associated with decreased levels of healthcare utilisation (advanced imaging, additional physician visits, lumbar spine injections, major surgeries, opioid medication use) and total medical costs when compared to delayed access.
Gellhorn et al. [35]	Retrospective cohort	Patients (general population) who received physician outpatient billing claims relating to lower back pain, sampled from the Centres for Medicare and Medicaid Services (*N* = 431,195; mean age = 76 years). Note: patients enrolled in comparative groups share similar population demographic characteristics, index diagnoses, comorbidities, and hospitalisations.	**Early access** (acute): Patients received physical therapy < 4 weeks following index physician visit. **Normal access** (subacute): Patients received physical therapy 4–12 weeks following index physician visit. **Delayed access** (chronic): Patients received physical therapy 3–12 months following index physician visit.	Lower risk of later medical service usage among patients who received physical therapy early after a back pain episode when compared to individuals who received physical therapy at later time points. Early physical therapy was strongly associated with decreased use of lumbosacral injections, physician office visits for low back pain, and lumbar surgery, when compared with physical therapy that occurred at later times. The authors also reported a positive dose–response relationship between time until physical therapy treatment commencement and risk of having to experience additional medical intervention.
Horn et al. [59]	Retrospective cohort	Patients (general population) with neck pain complaint presenting to physical therapy clinics in the U.S. (*N* = 1531; mean age of early treatment group = 46.2 ± 15.4 years, mean age of delayed treatment group = 52.4 ± 16.7 years).	**Early management:** Patients received physical therapy care < 4 weeks from self-reported symptom onset. **Delayed management**: Patients received physical therapy care > 4 weeks from self-reported symptom onset.	Early management was associated with increased odds of achieving clinical improvements that represented minimally clinically important differences (MCID) on the neck disability index (aOR = 2.01, 95% CI 1.57–2.56) and the numerical pain rating scale (aOR = 1.82, 95% CI 1.42–2.38) when compared to delayed management (reference group).
Hultman et al. [48]	Non-randomized interventional trial	Swedish patients (general population) presenting to a local hospital emergency department (*N* = 65; age range = 18–65 years)	**Early access (intervention) group**: Patients offered physiotherapy visits at 1–14 days (median = 4 days) following ED presentation. Follow-ups relating to outcomes measures were recorded at 3 weeks, 6 weeks, and 3 months. **Delayed access (control) group**: Patients contacted for follow-ups at 6 weeks and 3 months following ED presentation.	Early access group achieved significant increases (improvements) on the foot and ankle outcome score (FAOS) scale and on questions relating to self-evaluated physical activity and ankle function, at 6 weeks and 3 months, when compared to the delayed access group. No differences between groups were observed in clinical measures relating to joint range of motion, weight-bearing activity, or postural control.
Kucera et al. [56]	Case-control	Union-affiliated carpenters’ compensation claims related to back injuries in Washington State (U.S.); *N* = 4241.	**Early access:** Medical care initiated in less than 30 days from date of injury **Delayed access:** Medical care initiated 30 days or more after date of injury.	A delayed return to work after back injury (>90 days of paid lost work time) was more likely if there was a ≥30-day delay to accessing medical care than when access to medical care occurred sooner (aOR 3.6, 95% CI 2.1–6.1)
Nordeman et al. [37]	Randomized Controlled Trial	Patients (general population) presenting to primary healthcare centers in Sweden with lower back pain (*N* = 60; mean age of early treatment group = 39.2 ± 12.1, mean age of delayed treatment (control) group = 40.8 ± 11.1).	**Early access**: Patients received physiotherapy within two days of enrolment in study **Control, delayed access**: Patients received physiotherapy treatment after 4 weeks following enrolment in study	No significant differences in pain were reported between the groups at discharge. At 6 months of follow-up, pain was significantly lower in the early access group compared to the control group (*p* = 0.025); however, there were no differences in long-term disability, sick leave, or functional assessment.
Park et al. [51]	Randomized Controlled Trial	Patients (general population) presenting to a South Korean General Hospital with lateral epicondylitis (*N* = 31; mean age = 50 years)	**Early access:** Patients received treatment intervention immediately **Delayed access:** Patients received the treatment intervention after a 4-week period	Early access cohort had significantly greater improvements in pain levels when compared to the delayed group (*p* < 0.01) at 1 month follow-up time-point. There were no significant differences observed between the groups at 3-, 6-, or 12-month follow-up time points.
Rhon et al. [50]	Retrospective cohort	Individuals receiving care for ankle sprains within the U.S. Military Health System (*N* = 6150)	The study largely used a statistical approach that did not employ clear cut-points relating to early and delayed rehabilitation. Delay in commencing rehabilitation was instead calculated as a daily effect of each day that passed. One portion of the analysis utilised the following cut-points: **Early access:** Individuals received care within 4 weeks. **Delayed access:** Individuals received care within 8–12 weeks.	Receiving delayed rehabilitation increased the odds of a recurrence of ankle sprain when compared to earlier rehabilitation (OR = 1.28, 95% CI 1.10, 1.49). Compared to individuals receiving rehabilitation within 4 weeks, the odds of ankle sprain recurrence in individuals who received rehabilitation between 8–12 weeks were substantially higher (OR = 1.97, 95% CI not reported). Individuals receiving delayed rehabilitation care had greater odds of requiring additional rehabilitation (medical) visits (OR = 1.22, 95% CI 1.16, 1.27). With each additional day of delay in receiving rehabilitation care, there was a linear increase in the associated total treatment costs (OR 1.13, 95% CI 1.10, 1.17).
Rosenfeld et al. [39]	Randomized Controlled Trial	Patients (general population) presenting to primary healthcare centres in Sweden with acute whiplash injuries (*N* = 89)	**Early access:** Treatment provided within 96 h of injury **Delayed access**: Treatment provided after 14 days following injury	There were no significant differences in individual outcome measures associated with early versus delayed access to treatment at 6-month or 3-year follow-up, with passage of time from injury time-point the sole factor associated with pain level and measures of range of motion. However, when considering time to access treatment and treatment type in combination, only early active treatment achieved total cervical ranges of motion 3 years subsequent to their neck injury that matched those of uninjured controls. Those who received delayed access to treatment continued to have reduced cervical range of motion at that 3-year time point.
Rosenfeld et al. [40]	Prospective randomized trial	Patients (general population) presenting to primary healthcare centres in Sweden with acute whiplash injuries (*N* = 88; mean age of early treatment group = 32, mean age of delayed treatment group = 38).	**Early access:** Treatment provided within 96 h of injury **Delayed access**: Treatment provided after 14 days following injury	There were no significant differences in outcomes for early compared to delayed groups, with passage of time following injury the only factor associated with pain levels and measures of range of motion.
Rundell et al. [34]	Prospective cohort	Patients (general population) presenting to primary healthcare settings for a new back pain visit (*N* = 3705; age ≥ 65 years). Note: This population is outside the active service age for military service members. However, physical function of this age group has a strong bearing on areas of veteran health service delivery.	**Early access:** Physical therapy initiated ≤ 28 days from index physician visit **Delayed access**: No form of treatment provided until >28 days from index physician visit	There were no or marginal differences in pain, functional measures, and health-related quality measures at the 3-, 6-, or 12-month follow-up time-points among those who received early access when compared to the matched delayed access group. However, the early access group did have higher odds of improved function at the 12-month time-point (measured via Roland-Morris Disability Questionnaire) when compared to the matched group (OR 1.58, 95% CI 1.04–2.40). NOTE: Total actual received number of physical therapy sessions among the early group was highly variable.
Sohil et al. [32]	Retrospective cohort	Patients (general population) presenting to hospital emergency departments (ED) in Singapore for neck and back pain complaints (*N* = 125)	**Early access:** Patients received early physiotherapy evaluation and treatment (EPET) at a median of 4 days from index ED visit. **Delayed access:** Patients received standard care (SC) at a median of 34 days from index ED visit.	Patients in the early access (EPET) group had significantly lower levels of neck disability (9.0% vs. 33.4%, *p* < 0.001; measured via the neck disability index questionnaire) and pain (median value 1 vs. 4 points, *p* < 0.001; measured via the Modified Oswestry Low Back Pain Disability Questionnaire (MODI)) than delayed access (SC) patients (mean delay in treatment of 34 ± 22 days).
Wand et al. [38]	Single-blinded Randomized Controlled Trial	Patients (general population) presenting to primary physician care (i.e., general practitioners or emergency department) in London (England) for acute low back pain (*N* = 102; 35 ± 8.5 years).	**Early access:** Patients received immediate physiotherapy treatment following baseline assessment. **Delayed access:** Patients received treatment at 6 weeks from baseline assessment.	Early access patients demonstrated significantly better levels of disability, mood, general health, and quality of life compared to the delayed access group (*p* < 0.05) at 6-weeks of follow-up. At longer-term follow-up (i.e., >3 months) there were no significant differences between groups in the primary outcome measures of disability and pain. However, early access group patients did exhibit significantly less anxiety, depressive symptoms, and distress outcomes; and greater ratings of general, mental and emotional health.
Young et al. [49]	Retrospective cohort	Individuals receiving care for patellofemoral pain within the U.S. Military Health System (*N* = 74,408)	**Early access cohort one (i.e., first):** Individuals received physical therapy on the same day as diagnosis **Early access cohort two (i.e., early):** Individuals received physical therapy between 1 and 30 days after initial diagnosis **Delayed access:** Individuals received physical therapy between 31 and 90 days after initial diagnosis	Reduced odds of requiring additional healthcare (e.g., medical imaging, prescription medications, medical injections) for the diagnosed condition were observed in the early access cohorts (aORs * = 0.09–0.61) when compared to the delayed access cohort (aORs * = 1.64–2.20) [reference for calculation of aORs appears to have been overall cohort]. 2-year total health care costs for patellofemoral pain were significantly lower in the early access cohorts than in the delayed cohort. Odds of injury recurrence were higher in the delayed access cohort (aOR * = 1.78, 95% CI 1.36–2.33) than in the early access (first) cohort (aOR^*^ = 0.55, 95% CI 0.37–0.79). * Statistically significant (*p* < 0.05).
Zigenfus et al. [41]	Case-control	Workers’ cases of acute low back disorders were extracted from an occupational health care provider database (*N* = 3867; mean age of early treatment cohort = 35.1 ± 10.4 years, mean age of delayed treatment cohort (1) = 36.4 ± 10.8 years, mean age of delayed treatment cohort (2) = 36.9 ± 11.4 years)	**Early access:** Workers had an initial physical therapy session within ≤ 1 day of the injury (i.e., day of, or day after initial injury) **Delayed access cohort one:** Workers had an initial physical therapy session 2–7 days following injury **Delayed access cohort two:** Workers had a physical therapy session 8–197 days following injury	Early access workers experienced significantly lower numbers of physician visits (*p* < 0.01), injury case durations (*p* < 0.01), durations of restricted work (*p* < 0.01), and days away from work (*p* < 0.05) compared to both of the delayed access cohorts.

OR: Odds Ratio; aOR: Adjusted Odds Ratio; ED: Emergency Department; SC: Standard Care; EPET: Early Physical Therapy Evaluation and Treatment; MODI: Modified Oswestry Low Back Pain Disability Questionnaire; ODI: Oswestry Disability Index; FAOS: Foot and Ankle Outcome Score: MCID: Minimal Clinically Important Difference; LBP: Lower Back Pain.
